# mSphere of Influence: The discovery of a missing link in bacterial cell envelope biogenesis

**DOI:** 10.1128/msphere.00631-23

**Published:** 2024-02-01

**Authors:** Gregory A. Harrison

**Affiliations:** 1 Department of Molecular Biology and Microbiology, Tufts University School of Medicine, Boston, Massachusetts, USA; Washington University in St. Louis School of Medicine, St. Louis, Missouri, USA

**Keywords:** Gram-negative bacteria, outer membrane, phospholipids, AsmA-like proteins

## Abstract

Gregory Harrison is a bacteriologist researching essential pathways in bacteria as potential therapeutic targets. In this mSphere of Influence article, he reflects on a series of studies that employ complementary genetic approaches to define the crucial role of AsmA-family proteins in transporting phospholipids between the inner and outer membranes of Gram-negative bacteria. The authors of these three studies identify this family of lipid transporters through the means of bacterial genetics, answering a long-standing question in bacterial physiology, and serving as a reminder that a well-designed genetic strategy can go a long way in uncovering new biology.

## COMMENTARY

The pathways that generate the bacterial cell envelope are some of our most effective targets for antimicrobial development. The assembly and maintenance of the bacterial cell envelope is critical for bacterial growth and cell expansion as well as establishing a barrier against insults from the external environment. Deciphering the mechanisms underlying envelope biogenesis is fundamental to our understanding of bacterial physiology and our ability to develop new antimicrobials that target this essential process.

The architecture and composition of the envelope differs between bacterial species, but diderm (typically Gram-negative) species such as *Escherichia coli* harbor an outer membrane in addition to the plasma membrane that surrounds the cytosol. Unlike the cytoplasmic membrane that is composed of phospholipids, the outer membrane of diderms like *E. coli* contains both phospholipids and lipopolysaccharides (LPS). Both of these molecules are synthesized in the inner membrane and therefore must cross the periplasmic space to insert into the outer membrane. Decades of work on LPS have established that its transporter is a multiprotein complex that bridges the membranes called the Lpt system (1). Lpt has a hydrophobic groove that protects the lipid moiety of LPS molecules as they are pumped across the periplasm to the outer membrane (2–4). However, until very recently, it remained unclear how phospholipids were transported across the periplasm to the outer membrane. While my own research is not focused on outer membrane biogenesis, the story of how this mystery was unraveled serves as an elegant example of bacterial sleuthing using genetics. For this reason, I have chosen to reflect on three recent studies that tackled this long-standing research question (5–7).

In the first study, Grimm et al. took advantage of a mutant defective in outer membrane homeostasis (5). To fully appreciate the ingenuity of their approach, it is important to note that, like related Gram-negatives, the *E. coli* outer membrane is asymmetric, with LPS in the outer leaflet and phospholipids in the inner leaflet (8, 9). The uniform layer of LPS in the outer leaflet establishes a robust barrier, and mislocalized phospholipids in the outer leaflet disrupt the barrier making bacteria susceptible to external insults (10). *E. coli* encodes multiple mechanisms to remove these phospholipids and restore the membrane asymmetry (10, 11). One of these mechanisms is a transporter system called the maintenance of lipid asymmetry (Mla) pathway, which removes phospholipids from the outer leaflet and transports them back to the inner membrane (10). Previous work found a specific mutation in *mlaA* that causes the pathway to function in reverse (12). This mlaA* mutant has a stationary phase lysis phenotype, where the aberrant flow of phospholipids to the outer membrane causes the inner membrane to shrink and ultimately lyse. The authors exploited this lysis phenotype of mlaA* to select for transposon mutations that allow *E. coli* to stall stationary phase lysis (5). They discovered that deletion of *yhdP* likely decreases the flow of phospholipids from the inner membrane to the outer membrane. Thus, the authors concluded that YhdP promotes phospholipid transport to the outer membrane, although its precise mechanism remained unclear.

YhdP has similarity to the protein AsmA, and *E. coli* encodes a total of six AsmA-like proteins. In the next study, Ruiz et al. undertook an extensive reverse genetic approach to systematically delete the genes encoding each of the six AsmA-like proteins individually and in combination to test for functional redundancy (6). While all the single mutants and most double mutants had mild phenotypes at best, the Δ*yhdP* Δ*tamB* double mutant became extremely sensitive to environmental insults such as bile salts. In their efforts to create higher order mutants, Ruiz et al. discovered that they could delete up to five of the six AsmA-like proteins in a single mutant, and the bacteria survived so long as they had one functional copy of *yhdP*, *tamB*, or a third gene in this family, *ydbH*. They found that the AsmA-like proteins YhdP, TamB, and YdbH are redundant. Using bile salts to selectively inhibit growth of the Δ*yhdP* Δ*tamB* strain, Ruiz et al. isolated suppressor mutants that recover the ability to grow in the presence of this external insult. Remarkably, the suppressors each harbored a gain of function mutation in *ydbH*, consistent with their model that these three proteins function redundantly to establish outer membrane integrity. This functional redundancy likely explains why the role of this protein family remained elusive for so many years.

Notably, structural modeling of the AsmA-like proteins revealed that these transmembrane proteins form long channels of repeating taco-like domains that likely project into the periplasm. These channels contain a hydrophobic groove reminiscent of Lpt, strongly suggesting that YhdP, TamB, and YdbH are the long-sought phospholipid transporters in *E. coli*.

However, up to this point, the model was largely based on phenotypes associated with outer membrane integrity such as cell lysis and bile salt sensitivity. Phospholipid transport had not been measured directly. In the third study, Douglass et al. provided this final piece of the puzzle. To understand mechanisms to enhance bacterial sensitivity to the outer membrane targeting antibiotic polymyxin, Douglass et al. used transposon-insertion sequencing to identify mutants that become hypersensitive to polymyxin (7). Serendipitously, one of their hits was YhdP. Through a series of elegant reverse genetic experiments, similar to Ruiz et al., the authors independently reach the conclusion that the AsmA-like proteins YhdP, TamB, and YdbH are redundant, and *E. coli* requires at least one of them to survive. Motivated by outer membrane defects observed in their study, they monitored the synthesis and distribution of lipids in a mutant lacking both TamB and YdbH and found that phospholipids in this double mutant were trapped in the inner membrane and could not traffic efficiently to the outer membrane. This study provided the necessary direct biochemical evidence that AsmA-like proteins are specifically required for phospholipid transport.

These studies highlight the power of classic genetic techniques to tackle the unknown. Each study used an innovative forward genetic strategy. However, the functional redundancy of these proteins complicated these approaches, since the phenotype of any single mutant is subtle and might easily be overlooked. Were it not for the herculean effort to generate higher-order mutants lacking the AsmA proteins in dozens of combinations, the essential role of this protein family in phospholipid transport might still remain obscure. Ultimately, these studies have taught me not to shy away from complicated questions and encouraged me to embrace subtle or unexpected phenotypes as an indication that something deeper might be going on.

## AFTER EPUB

*[This article was published on 1 February 2024 with an error in a protein name. The text was corrected in the current version, posted on 14 February 2024.]*
